# Supplementary material for: Social determinants of health in lesbian, gay, bisexual, transgender, queer, and other sexual and gender minority (LGBTQ+) older adults: Impact of socioeconomic disadvantage on inpatient hospitalizations
Source: PLoS One. 2025 Sep 3;20(9):e0330612. doi: 10.1371/journal.pone.0330612 (PMC12407415; doi:10.1371/journal.pone.0330612)
Supplement: Attachment — Submitted filename: Response to Reviewers.docx [file pone.0330612.s003.docx]

**Reviewer Comments**

**Reviewer #1**

1. The review has addressed all of the reviewers' concerns and queries. The authors did a competent job in refining this submission.

*Thank you!*

**Reviewer #2**

1. A majority of the original comments were addressed. The manuscript is technically sound, the data support the conclusion, and the analyses were appropriate and rigorous. The authors indicate the data cannot be shared publicly because of data privacy, but is available upon request. However, minor revisions are recommended as there are still a large number of grammatical errors in the text that were not addressed.

*Thank you!*

2. It is recommended the authors correct the following errors and review their manuscript carefully for any additional grammatical errors missed by the reviewer:

*I appreciate the level of detail the errors are listed and the opportunity to correct these. I apologize for the oversight. We have carefully reviewed and provided an additional edit to correct the font of the reference page.*

Line 165: “…or it can be entered it in the clinical setting…” to “…or can be entered in the clinical setting…”

*Corrected.*

Line 195-196: “…when there is a significant overall effect was detected.” to “…when a significant overall effect was detected.”

*Corrected.*

Line 243: “…the majority of (66.4%) of hospitalized LGBTQ+…” to “…the majority (66.4%) of hospitalized LGBTQ+…”

*Corrected.*

Line 276: “…backgrounds are more likely to receive support from multiple sources…” to “…backgrounds being more likely to receive support from multiple sources…”

*Corrected.*

Line 287: typo—change “my” to “may”

*Corrected.*

Line 291: typo—change “tavel” to “travel”

*Corrected.*

Line 298: typo—change “my” to “may”

*Corrected.*

Line 299: typo—change “aniti-discrimination” to “anti-discrimination”

*Corrected.*

Line 350-351: “Street level data would would have…” to “Street level data would have…”

*Corrected.*

Line 362: “…particularly or older…” to “…particularly for older…”

*Corrected.*

Line 364: “Since the pandemic there…” to “Since the pandemic, there…”

*Corrected.*

Line 366: “Depending on which disadvantage index used…” to “Depending on which disadvantage index is used…”

*Corrected.*

3. Except for these grammatical issues, the manuscript is well-written and engaging. In particular, the Discussion, Future Directions, and Limitations sections were well executed and robust.

*Thank you!*
