# Supplementary material for: Social determinants of health in lesbian, gay, bisexual, transgender, queer, and other sexual and gender minority (LGBTQ+) older adults: Impact of socioeconomic disadvantage on inpatient hospitalizations
Source: PLoS One. 2025 Sep 3;20(9):e0330612. doi: 10.1371/journal.pone.0330612 (PMC12407415; doi:10.1371/journal.pone.0330612)
Supplement: Attachment — Submitted filename: PLOS One Response to Reviewer Comments.docx [file pone.0330612.s002.docx]

Please find the reviewer’s comments and our responses below. Thank you for this opportunity to revise and resubmit.

1. **Justify the Exclusion of Emergency Room Visits**

*The Introduction references prior research on socioeconomic status and hospital/emergency room utilization. However, the Methods section does not clarify why emergency room visits were excluded. The authors must provide a strong rationale for this exclusion, as ER visits are a critical component of healthcare access, particularly in socioeconomically disadvantaged populations.*

**Authors’ comments:** Thank you for the comment. We agree that ED visits are a critical component of care and hope to look at this with future research studies. The focus of the study was on inpatient hospitalizations only. In the introduction the Armenia et al. (2017) article describes an observational study from the National Inpatient Sample database of the Healthcare Cost and Utilization Project (HCUP) data. The study discusses emergency surgeries of patients who are admitted as inpatient status. We have clarified throughout the manuscript by using the term “inpatient” before hospitalizations where appropriate, including the title.

2. **Clarify the Relationship Between ADI Groups and Primary Diagnoses/Hospitalizations per Patient**

*The Results section does not explicitly explore whether there is a correlation between ADI groups and specific health conditions or the frequency of hospitalizations per patient. If no relationships were found, this should be clearly stated. If such data exist but were not analyzed, the authors should consider including it, as it would enhance the study’s clinical relevance.*

**Authors’ comments:** We agree that exploring the relationship between ADI group and specific health conditions or frequency of hospitalization per patient would be useful. However, the study was not part of the information extracted from the electronic medical records. Thus, we did not conduct these analyses.

3. **Provide References for Social Support and Community Connection Claims**

*The Discussion section states that differences in sexual orientation across ADI groups suggest differences in social support and community connection. However, no references are provided to support this claim. The authors must either cite relevant literature or provide empirical justification for these assertions.*

**Authors’ comments:** We have added supporting information for this statement. The additional information reads: Pg 14, lines 267-272 - This is supported by research showing that socioeconomic status (SES) significantly impacts social support networks among LGBTQ+ individual, with higher SES individuals from higher socioeconomic backgrounds are more likely to receive support from multiple sources, including family, peers, and significant others, while those from lower socioeconomic backgrounds often lack family support and experience worse mental health outcomes.[31, 32]

**Soft Recommendations**

1. **Improve Writing and Grammar Throughout the Manuscript**

*The manuscript contains multiple spelling and grammatical errors, as noted by Reviewer 2. Specific lines (99-101, 163-166, 192-193, etc.) require correction, but the entire manuscript should be reviewed for clarity and coherence. A professional language edit is strongly recommended.*

**Authors’ comments:** Thank you for bringing these errors to our attention.

Line 57 – correction to the spelling of “identity”

Line 65 - correction to the spelling of “county”

Lines 97: We have added a comma after the word “model” in line 97 to now read as: “Guided by the social ecological model,[19]…”. This aligns with the Vancouver style of intext citations.

Line 100 the spelling of incapsulated has been corrected to “encapsulated”.

2. **Clarify the Placeholder Citation ("XX et al. 2023[21]")**

*On line 165, there is a reference to “(see XX et al. 2023[21]).” If this is an intentional blinding of author names for review purposes, it should be indicated. If it was left in by mistake, it must be corrected.*

**Authors’ comments:** Thank you. We have inserted “blinded for review” as a placeholder and have added this to the references list.

3. **Discuss the Potential for Future Comparisons to Heterosexual and Cisgender Counterparts**

*While the Introduction makes comparisons between LGBTQ+ individuals and their heterosexual/cisgender counterparts, the Discussion does not return to this point. The authors should consider acknowledging this as a potential future research direction.*

**Authors’ comments:** Thank you for this suggestion. Pg 17, lines 330-335 reads: While some studies might benefit from comparing LGBTQ+ and non-LGBTQ+ populations, it’s equally important to conduct research that explores the rich diversity within LGBTQ+ older adult communities without necessarily using non-LGBTQ+ populations as a reference point. Longitudinal studies examining how health experiences and needs evolved over time could provide important information for developing long-term strategies to promote equity in aging populations.
